# Carbapenemase-Producing Escherichia coli: Comparison of a Novel Rapid Lateral Flow Assay With the Polymerase Chain Reaction (PCR) and Antimicrobial Resistance Pattern

**DOI:** 10.7759/cureus.68941

**Published:** 2024-09-08

**Authors:** Sharon Christina, Raveendran Praveena, Mymoonah Risha Shahul, Chitralekha Saikumar

**Affiliations:** 1 Microbiology, Sree Balaji Medical College & Hospital, Bharath Institute of Higher Education and Research, Chennai, IND

**Keywords:** antimicrobial resistance, carbapenemase-producing escherichia coli, knivo detection k-set, lateral flow assay, pcr

## Abstract

Background

In critically ill patients, carbapenems are often used as the last line of treatment. Carbapenem-resistant *Enterobacterales* (CRE) present an extreme challenge to treatment due to their resistance to various antibiotics. Optimal therapy for patients and infection control relies on the early and accurate diagnosis of these infections. The K.N.I.V.O. Detection K-Set is a newly developed immunological rapid test developed to identify the presence of carbapenemase in Gram-negative bacteria resistant to multiple drugs.

Objectives

This study evaluates a new K.N.I.V.O. Detection K-Set and its application for the rapid detection of isolates of multidrug-resistant *Escherichia coli* (MDR *E. coli*) that produce carbapenemase. This test aims to compare the test's performance to the polymerase chain reaction (PCR) method.

Methods

The study included 150 MDR *E. coli* isolates that were confirmed to be resistant to at least three groups of antibiotics, including carbapenems. The test followed the manufacturer's instructions using the K.N.I.V.O. Detection K-Set. The outcomes were compared with carbapenemase gene detection (bla-KPC, bla-NDM, bla-OXA-48, bla -VIM, and bla -IMP) using the PCR. The K-Set's sensitivity, specificity, positive predictive value (PPV), and negative predictive value (NPV) were calculated and studied.

Results

The K.N.I.V.O. Detection K-Set showed highly effective diagnostic performance with a 97.1% sensitivity, 97.5% specificity, 97.1% positive predictive value, and 98.7% negative predictive value. Seventy-eight of the 150 isolates were proven to be producers of carbapenemase, with 68 of those cases having an accurate identification. The remaining isolates were found to be non-producers. Within 15 minutes, the rapid test provided results.

Conclusion

The K.N.I.V.O. Detection K-Set is an effective and rapid method for identifying carbapenemase producers among MDR *E. coli* isolates. Its rapid processing time, associated with its high sensitivity and specificity, indicates that it can increase the effectiveness of diagnostic laboratories and better patient care in clinical settings. Implementing such rapid screenings could be vital for controlling the spread of drug-resistant infections and enhancing antimicrobial stewardship. This also ensures that patients receive timely treatment and effective care.

## Introduction

*Escherichia coli* (*E. coli*) is the most common Gram-negative bacteria that causes hospital- and community-acquired bloodstream infections (BSIs), which is clinically significant. It is also a foremost cause of death in people of all age groups [1]. Nosocomial infections, such as ventilator-associated pneumonia and urinary tract infections (UTIs), are primarily caused by *E. coli* [2]. It also causes diarrhea. Public health concerns are being raised globally by the emergence and spread of antibiotic resistance. The prevalence of antibiotic resistance in *E. coli* strains is rising, resulting in a range of intestinal disorders [3].

In addition to increasing antibiotic resistance, which leads to limited therapeutic options and high mortality rates, carbapenemase-producing *Enterobacteriaceae* (CPE) presents an immediate threat to worldwide public health [4]. Early diagnosis is essential for effective treatment response and the implementation of infection control measures [5].

The decreased susceptibility of *Enterobacteriaceae* to carbapenems can be associated with different mechanisms. These include the production of carbapenemase and beta-lactamase enzymes with significant hydrolytic activity against carbapenems or overexpression efflux pump in combination with beta-lactamases with weak carbapenemase activity.

In addition, decreased outer-membrane permeability due to the action of other multidrug-resistant genes, such as those targeting cephalosporins and extended-spectrum beta-lactamases also plays a role [6]. Most of the clinically relevant carbapenemases are Ambler class A (mostly KPC-type), Ambler class B (metallo-beta-lactamases (MBLs) including IMP, VIM, and NDM types), and Ambler class D (enzymes similar to OXA-48) [7].

Antibiotic susceptibility testing was performed using routine conventional methods, the disc diffusion method on Mueller-Hinton agar obtained from Himedia, Mumbai, Maharashtra, India, as well as the automated VITEK® 2 COMPACT system (bioMérieux, Inc. Durham, NC). Multidrug-resistant *E. coli* (MDR *E. coli*) strains and the carbapenemase-producing *E. coli* among them were identified. Furthermore, the gene responsible for resistance is identified using the polymerase chain reaction (PCR) (Eppendorf Germany) [8].

Highly accurate, standard detection techniques like PCR need specialized equipment and skilled workers and are time-consuming [9]. In resource-limited situations, rapid diagnostic techniques, especially immunological assays, may be an alternative for the rapid identification of CPE.

Carbapenem-resistant K.N.I.V.O. Detection K-Set Bio-State Inc. obtained from Haryana, India, is a lateral flow assay test for qualitatively identifying several carbapenemase types in bacterial colonies. It works on the principle of sandwich immunochromatographic tests. It is a new immunological rapid test that identifies carbapenemase producers from bacterial cultures immediately. This novel immunochromatographic test detects five major carbapenemases: KPC, NDM, IMP, VIM, and OXA-48-like enzymes [10].

## Materials and methods

A total of 150 MDR *E. coli* was isolated from clinical samples received at the Department of Microbiology of Sree Balaji Medical College & Hospital, a tertiary care hospital in Chennai, India, for a period of one year from January to December 2023. The isolates were identified by routine microbiological culture methods. They were found to be multidrug resistant by the Kirby-Bauer disc diffusion method and VITEK® 2 COMPACT based on their resistance to at least three or more classes of antibiotics, including carbapenems. It was confirmed as carbapenemase-producing *E. coli* and analyzed using the PCR and the K.N.I.V.O. Detection K-Set.

The K-Set kit consists of two test cassettes: cassette A detects the genes NDM, KPC, and IMP, while cassette B identifies VIM and OXA-48-type [11].

If the carbapenemase of the KPC, NDM-, IMP, VIM, or OXA-48 types are present in the specimen, they bind to the gold-conjugated anti-KPC, anti-NDM, anti-IMP, anti-VIM, or anti-OXA-48 antibodies.

The antibody-antigen complex wicks up the membrane, interacting with test lines that have immobilized monoclonal antibodies. This forms a visible line for the test. Proper flow and reagent reactivity ensure the gold-conjugated antibodies move to the control line, making it visible.

The test was performed following the manufacturer's guidelines. Five drops of the sample treatment solution were taken in a test tube. A single-use inoculation loop was inserted into the colony and dipped into the solution. A vortex was done to combine the mixture. Cassettes A and B were placed side by side, and then 50 µL of the mixture was poured into each sample hole. The results were read and recorded after 10 to 15 minutes.

A single red line appearing in the control line region (C) indicates a negative result. The control line acts as an internal quality control. If it does not appear, the result is invalid. The presence of one or more red lines in the test area, in addition to a red line in the control line region (C), indicates a positive result for the related genotype carbapenemase. If the control line is absent and the test line is red, the test result is invalid [12].

A PCR test was performed to identify specific DNA sequences of carbapenemase genes in bacteria. Using specific primers, the target DNA regions were amplified, allowing for the precise identification of genes responsible for carbapenem resistance. The genomic DNA of the *E. coli* isolates was separated. Genomic DNA from the *E. coli* isolates was extracted using a commercial DNA extraction kit. For the PCR setup, the PCR mix was prepared, including a PCR buffer at a 1x concentration, a dNTP mix with a final concentration of 200 µM for each dNTP, specific primers targeting carbapenemase genes (KPC, NDM, VIM, IMP, and OXA-48) with a final concentration of 0.2-0.5 µM for each primer, 1-2 units of Taq DNA polymerase, 1-10 ng of genomic DNA as the template, and nuclease-free water to bring the reaction volume to 25-50 µL. Primers were designed specifically for the carbapenemase genes of interest, with provided primer sequences

The PCR conditions included an initial denaturation at 94°C for five minutes, followed by 30-35 cycles of denaturation at 94°C for 30 seconds, annealing at 50-60° for 30 seconds, and extension at 72°C for one minute, with a final extension at 72°C for five to 10 minutes. PCR products were then run on a 1.5-2% agarose gel containing a DNA stain to visualize the amplified DNA fragments. A DNA ladder to estimate the size of PCR products, and the gel was visualized under UV light and fixed using a gel documentation system. The size of the PCR products was compared with the expected sizes of the carbapenemase gene amplicons, with the presence of bands at the correct sizes indicating the presence of the respective carbapenemase genes. Positive controls were included to ensure the PCR reaction was working properly, and negative controls, including a no-template control (NTC), were included to check for contamination.

The Institutional Human Ethics Committee of Sree Balaji Medical College & Hospital, Bharath Institute of Higher Education & Research, Chennai issued approval (no. 002/SBMCH/IHEC/2024/2192).

## Results

Antimicrobial susceptibility testing was done using the Kirby-Bauer disc diffusion method and VITEK® 2 COMPACT, and the results were interpreted using the CLSI 2023 M 100 33rd edition. Notably, high resistance rates were observed for ampicillin 130 (86.7%), amoxicillin/clavulanic acid 120 (80.0%), and cefotaxime 125 (83.3%). The resistance rate for meropenem and imipenem was 67 (44.7%), indicating a significant resistance level. Other antibiotics like piperacillin/tazobactam and ciprofloxacin had 95 (63.3% )and 115 (76.7%), respectively. Lower resistance rates were seen with cefepime and amikacin 90 (60.0%), as shown in Table 1.

**Table 1:** Antibiotic resistance patterns for 150 multidrug-resistant Escherichia coli isolates

Antibiotic	Strength (µg)	No. of sensitive isolates	No. of Resistant isolates	Percentage Resistant (%)
Ampicillin	10	20	130	86.7
Amoxicillin/clavulanic acid	20/10	30	120	80
Cefazolin	30	40	110	73.3
Cefotaxime	30	25	125	83.3
Ceftriaxone	30	25	125	83.3
Gentamicin	10	40	110	73.3
Piperacillin/tazobactam	100/10	55	95	63.3
Ciprofloxacin	5	35	115	76.7
Cotrimoxazole	1.25/23.75	45	105	70
Cefuroxime	30	35	115	76.7
Cefepime	30	60	90	60
Ceftazidime	30	50	100	66.7
Imipenem	10	83	67	44.7
Meropenem	10	83	67	44.7
Tobramycin	10	55	95	63.3
Amikacin	30	60	90	60
Tetracycline	30	50	100	66.7
Aztreonam	30	35	115	76.7

**Figure 1:**
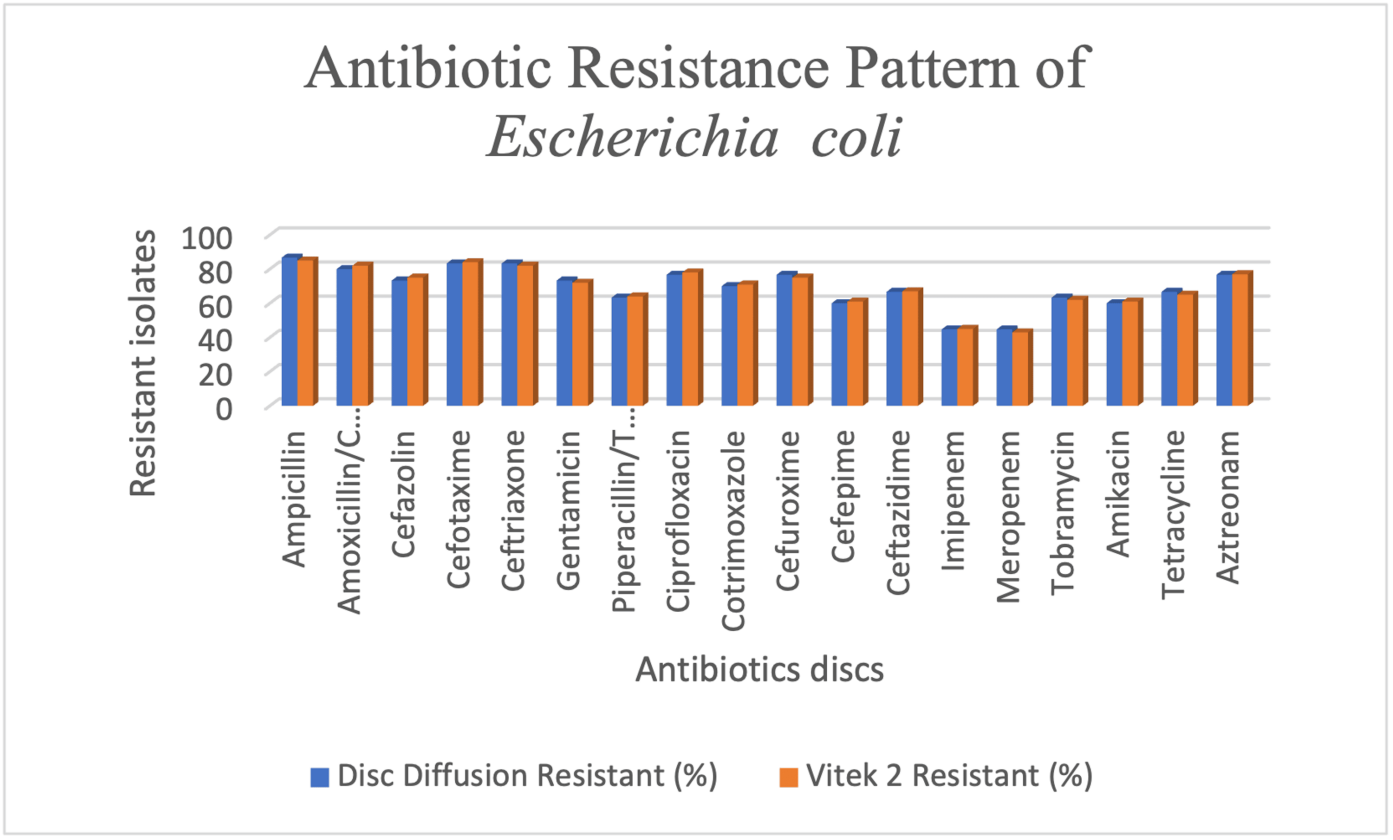
Antibiotic resistance pattern of Escherichia coli

Figure 1 shows the antibiotic resistance pattern of *E. coli *with disc diffusion and VITEK® 2 COMPACT.

**Figure 2:**
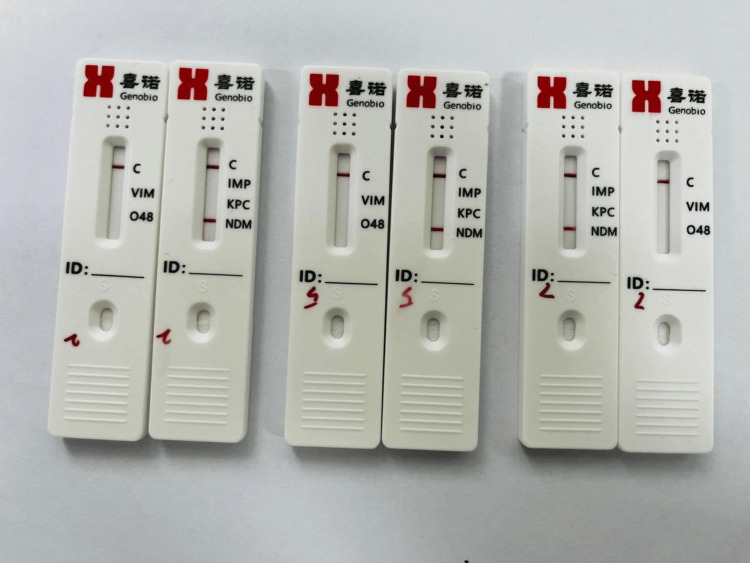
K.N.I.V.O. Detection K-Set showing a positive result for the NDM gene as a red line. Positive control also shows as a red line.

The performance of the K.N.I.V.O. Detection K-Set was evaluated against PCR results for detecting carbapenemase-producing *E. coli*. The following results, as shown in Table 2, identified the presence of the NDM gene in carbapenemase-producing *E. coli* isolates, providing clear positive results within 15 minutes, as shown in Figure 2.

**Table 2:** The K.N.I.V.O. Detection K-Set versus PCR in identifying carbapenemase-producing Escherichia coli.

Carbapenemase gene	K-Set No. of isolates (%) Identified	K-Set No. of isolates (%)Not identified	No. of isolates detected by PCR (%)
Positive	68 (45%)	2 (1.3%)	70 (47%)
Negative	78 (52%)	2 (1.3%)	80 (53%)
Total	146 (97.3%)	4 (2.6%)	150 (100%)

Out of 150 multidrug-resistant (MDR) *E. coli *isolates, 70 were observed to produce carbapenemase with the PCR. The rapid detection test identified 68 (45%) of the 70 (47%) carbapenemase producers and identified 78 (52%) out of 80 (53%) non-producers, resulting in a sensitivity of 97.1% and a specificity of 97.5%, as shown in Table 2.

The test showed a high sensitivity of 97.1% and specificity of 97.5%, providing the test's effectiveness in correctly identifying 97.5% of true-negative cases, thereby reducing the incidence of false positives. The positive predictive value (PPV) of 97.1% suggests that when the test indicates a positive result, there is a 97.1% probability that the individual truly has the condition. The negative predictive value (NPV) is remarkably high at 98.7%, as shown in Table 3. Together, these metrics show the test's reliability and accuracy in detecting and ruling out the condition.

**Table 3:** Performance metrics of K.N.I.V.O. Detection K-Set

Parameter	Percentage value (%)
Sensitivity	97.1
Specificity	97.5
Positive predictive value (PPV)	97.1
Negative predictive value (NPV)	98.7

## Discussion

Carbapenem-hydrolyzing enzymes are increasingly seen among isolates of *E. coli* as hospital- and community-acquired opportunistic pathogens, as a result of the worldwide distribution of carbapenemase-producing *Enterobacteriaceae* (CPE) strains [13]. Several phenotypic detection methods have been developed to identify and differentiate between the different types of carbapenemases and these methods can be utilized for carbapenem-resistant *Enterobacterales* (CRE) prevention

To identify the different types of carbapenemases, various phenotypic methods were evolved and can be used for CRE prevention [14].

A total of 150 MDR *E. coli* was identified using the disc diffusion method and automated system, Vitek 2. They were resistant to beta-lactamase inhibitors, cephalosporins, and carbapenems like imipenem and meropenem. There were slight variations between automated and manual testing approaches.

Table 1 shows the antibiotic resistance patterns of 150 isolates. It was seen that the highest percentage of resistance was observed against ampicillin (86.7%), followed by ceftriaxone and cefotaxime (83.3%) and amoxicillin/clavulanic acid (80%). A big portion (76%) of the *E. coli* isolates were resistant to ciprofloxacin and cefuroxime. Carbapenems showed antimicrobial resistance of 44.7% to both imipenem and meropenem.

As shown in Table 2, the carbapenemase producers and non-carbapenemase producers were 47% and 53% by the PCR and 45% and 52% by the rapid detection K-Set, respectively. Recent studies have shown the therapeutic and economic benefits of rapid diagnostic tests for identifying antibiotic resistance.

In 2021, Chavda et al.'s study revealed the value of early diagnoses in preventing CPE while improving healthcare for patients [15]. According to Ghaith et al., the evaluation of the carbapenem-resistant K.N.I.V.O Detection K-Set revealed that its specificity ranged from 93.5% to 100% and its sensitivity ranged from 96.5% to 100% [16].

Boutal H et al. found 100% sensitivity and 100% specificity in identifying NDM-like CPE using another lateral flow immunoassay [17]. In a study by Sadek et al., it is noteworthy that isolates that produce double carbapenemase, like KPC-2 and VIM-1, NDM-1, and OXA-48 kinds, might be found with this test. Among the isolates tested, there were no false positives. High percentages, i.e., 96.8% (CI95 93.6-98.4%) and 100% (CI95 79.6-100%), were determined to be the overall sensitivity and specificity, respectively [10]. According to Al Khamis M's study, the major resistances caused by carbapenemases produced by Enterobacteriaceae are the NDM and OXA-48 enzymes [18].

The present study also had high sensitivity and specificity for the K.N.I.V.O. Detection K-Set, which is similar to previous studies. It contributes to the pool of diagnostic equipment with quick results and ease of use that are available to combat antimicrobial resistance.

The appropriate use of antibiotics and the implementation of efficient infection control measures depend on the early detection of carbapenemase production, both of which can have serious effects on patient outcomes.

Rapid diagnostics has a high potential to reduce healthcare costs as in a study by Pierce et al., who estimated that the implementation of rapid diagnostics could save significant healthcare expenditures by reducing the length of hospital stays and the need for broad-spectrum antibiotics [11].

The K.N.I.V.O. Detection K-Set, with its high sensitivity and specificity, provides a valuable tool for the rapid detection of carbapenemase-producing *E. coli*. Its application in clinical settings can facilitate timely decision-making and improve infection control practices.

## Conclusions

The PCR can accurately identify the presence of specific carbapenemase genes, such as KPC, NDM, VIM, IMP, and OXA-48. This method identifies the genes causing antibiotic resistance rapidly and accurately, offering essential information for infection control and research. However, PCR requires specialized equipment and skilled personnel, which is challenging in resource-limited settings. With MDR *E. coli*, the K.N.I.V.O. Detection K-Set revealed reliable sensitivity and specificity for identifying carbapenemase producers, comparable to PCR results. Its rapid turnaround time and user-friendliness make it a useful tool for clinical laboratories.

The use of the K.N.I.V.O. Detection K-Set in clinical settings could aid in the management of infections caused by these resistant pathogens, improving infection control measures and patient outcomes.
